# STK17B promotes carcinogenesis and metastasis via AKT/GSK-3β/Snail signaling in hepatocellular carcinoma

**DOI:** 10.1038/s41419-018-0262-1

**Published:** 2018-02-14

**Authors:** Yaliang Lan, Jihua Han, Yan Wang, Jiabei Wang, Guangchao Yang, Keyu Li, Ruipeng Song, Tongsen Zheng, Yingjian Liang, Shangha Pan, Xirui Liu, Mingxi Zhu, Yao Liu, Fanzheng Meng, Manzoor Mohsin, Yifeng Cui, Bo Zhang, Sharma Subash, Lianxin Liu

**Affiliations:** 1 Department of Hepatic Surgery, The First Affiliated Hospital of Harbin Medical University, Key Laboratory of Hepatosplenic Surgery, Ministry of Education, Harbin, China; 2 Department of Gastrointestinal Medical Oncology, The Affiliated Tumor Hospital of Harbin Medical University, Harbin, China

## Abstract

Hepatocellular carcinoma (HCC) is a lethal malignancy worldwide with frequent intrahepatic and distant metastasis. Elucidating the underlying molecular mechanism that modulates HCC progression is critical for exploring novel therapeutic strategies. Serine/Threonine Kinase 17B (STK17B) is upregulated in HCC tissues, but its role in HCC progression remains elusive. In the present studies, we reported that STK17B had a critical role in HCC progression. STK17B was significantly upregulated in HCC cell lines and specimens, and patients with ectopic STK17B expression characterized with poor clinicopathological features. In vitro and in vivo assay demonstrated that inhibition of STK17B markedly inhibits HCC tumorigenesis and metastasis, while STK17B overexpression promoted these processes. Furthermore, we found that STK17B promoted EMT process via activating AKT/GSK-3β/Snail signal pathway, and miR-455-3p was identified as the upstream regulator of STK17B. Combination of high level of STK17B and low level of miR-455-3p predicted poor prognosis with higher accuracy for HCC patients. In conclusion, our research demonstrated that STK17B promotes HCC progression, induces EMT process via activating AKT/GSK-3β/Snail signal and predicts poor prognosis in HCC.

## Introduction

Hepatocellular carcinoma (HCC), the fifth most common malignancy globally, causes almost 0.7 million deaths annually, and HCC has become the third leading cause of cancer-related mortality^1^. Currently, HCC is still treated most effectively through surgical resection and liver transplantation although multiple novel therapeutic strategies have been developed. Furthermore, most HCC patients are diagnosed at an advanced stage, which precludes surgery as a feasible treatment option. Recurrence and metastasis after various treatments are the most intractable obstacles hindering a marked enhancement of the overall survival rate among HCC patients^2^. Over the past few decades, numerous basic research have been conducted on HCC, however, further investigation is still necessary to elucidate the mechanism underlying HCC carcinogenesis and thus develop an optimal therapeutic strategy for HCC.

Serine/threonine kinase 17B (STK17B), whose gene is located on chromosome 2 (2q32.3), was first reported by Sanjo et al. in 1998^3^. Previous findings have indicated that STK17B expression is highly enriched in B and T cells suggesting the possible involvement of this gene in immunological processes^4^. Therefore, STK17B has been identified as a promising therapeutic target for type 1 diabetes, multiple sclerosis, and graft rejection^5^. There are also some reports demonstrated that STK17B was related to apoptosis in various cell types, such as islet β-cells and acute myeloid leukemia cells^6,7^, but it is still controversial and its ability to regulate apoptosis seems to depend on the cellular context^8,9^. Some researches revealed that STK17B was deregulated in some cancers and have important role in cancer progression. Yang et al.^10^ found that endogenous STK17B expression was elevated in basal-like and HER2-enriched breast cancer, and loss of STK17B suppressed tumorigenesis and tumor growth in xenograft model. Hartmann et al.^11^ reported that STK17B was expressed at high levels in cutaneous T-cell lymphomas, and Tomimaru et al.^12^ showed that STK17B was overexpressed in HCC tissue. These findings indicated that STK17B might promote tumor progression. However, a consensus of STK17B’s function in cancer was not reached in previous studies, because STK17B may act as a tumor suppressor in leukemia and colorectal cancer^7,13^. Thus, the information indicated that STK17B may have dual functions in tumor progression, and we suspected that STK17B functions in a disease-dependent or cellular-dependent manner.

Epithelial–mesenchymal transition (EMT) is critical in the progression of metastasis in multiple cancers. In this process epithelial cells are induced to gain mesenchymal characteristic^14^. Various signaling pathways that were abnormally activated or inactivated in tumor progression were responsible for regulating EMT process, such as TGF-β/Smad, JAK/Stat3, and Wnt/β-catenin^15–17^. Previous studies have indicated that AKT/GSK-3β/Snail signal pathway is involved in metastasis by modulating EMT process^18–20^. In this pathway, activated AKT phosphorylates and inhibits GSK-3β, resulting in rescues of Snail, which is inhibited by GSK-3β. Then, Snail regulates EMT process as a transcriptional suppressor^21^.

Our aim here was to comprehensively investigate the precise function of STK17B in HCC progression and uncover its underlying mechanism of action. We obtained evidence indicating that STK17B is upregulated in the majority of HCC tissues, and demonstrated that STK17B overexpression stimulates HCC cell proliferation and metastasis both in vitro and in vivo. Furthermore, we found that STK17B is regulated by miR-455-3p, which was reduced in HCC, and that STK17B activates AKT/GSK-3β/Snail signaling and thereby induces the EMT process.

## Results

### STK17B is frequently upregulated in HCC and predicts poor prognosis

**Fig. 1:**
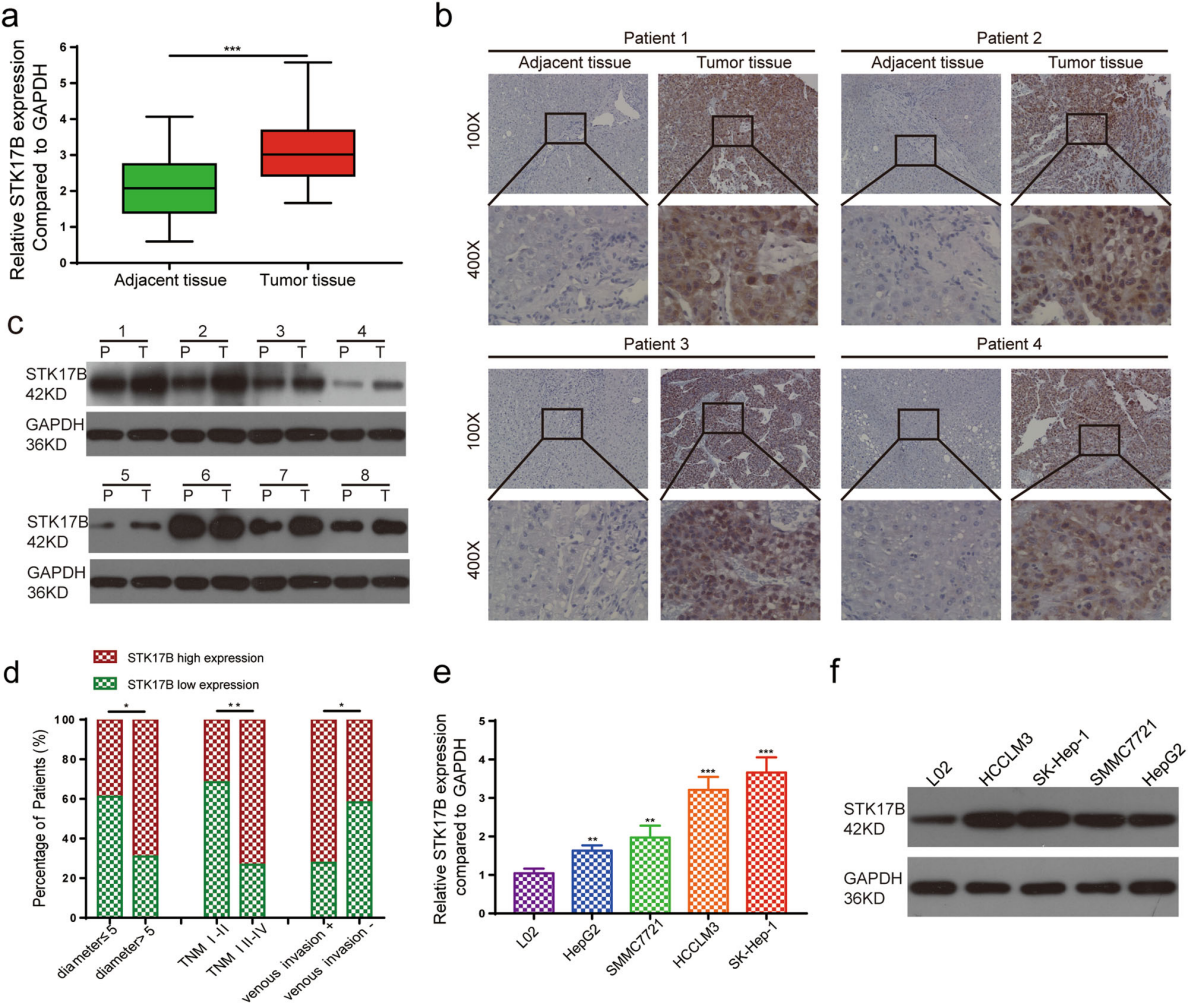
STK17B is upregulated in HCC and predicts poor clinicopathological feature. **a** STK17B was upregulated on mRNA level as determined by qRT-PCR using HCC tissue and corresponding ANLTs. **b** Representative IHC staining indicated that STK17B was upregulated in HCC tissue compared with ANLTs. **c** Western blotting analysis of STK17B expression in HCC tissues and matched ANLTs. **d** Upregulation of STK17B was associated with poor clinicopahtological feature, including tumor size, TNM stage, and venous invasion. **e** STK17B was overexpressed on mRNA level in HCC cell lines (HCCLM3, SK-Hep-1, HepG2, and SMCC7721) compared with normal liver cell L02. **f** Western blotting analysis suggested that STK17B was upregulated on protein level in HCC cell lines. GAPDH was used as internal control. The data are expressed as mean ± SD of three independent experiments. *P < 0.05; **P < 0.01; ***P < 0.001

We measured STK17B expression in 60 pairs of HCC tissue by using quantitative real-time polymerase chain reaction (qRT-PCR), which indicated that STK17B mRNA expression was higher in HCC tissues than that in adjacent non-tumorous liver tissues (ANLTs) (Fig. 1a). Accordingly, the results of immunohistochemistry (IHC) and western blotting analysis showed that STK17B protein was upregulated in HCC tissues (Fig. 1b, c). Moreover, clinicopathological analysis revealed that STK17B expression level was positively associated with tumor size (*P = *0.044), TNM stage (*P = *0.004), and venous invasion (*P = *0.031), indicating poor clinicopathological feature (Fig. 1d). We also measured STK17B levels in cell lines by performing qRT-PCR and western blotting. STK17B expression was elevated in HCC cell lines (HCCLM3, SK-Hep-1, HepG2, SMMC7721), but decreased in the normal liver cell line L02 (Fig. 1e, f). Together, these data indicated that STK17B upregulation is a frequent event in HCC tissues and cell lines.

### STK17B promotes HCC cell proliferation and tumorigenesis in vitro and in vivo

**Fig. 2:**
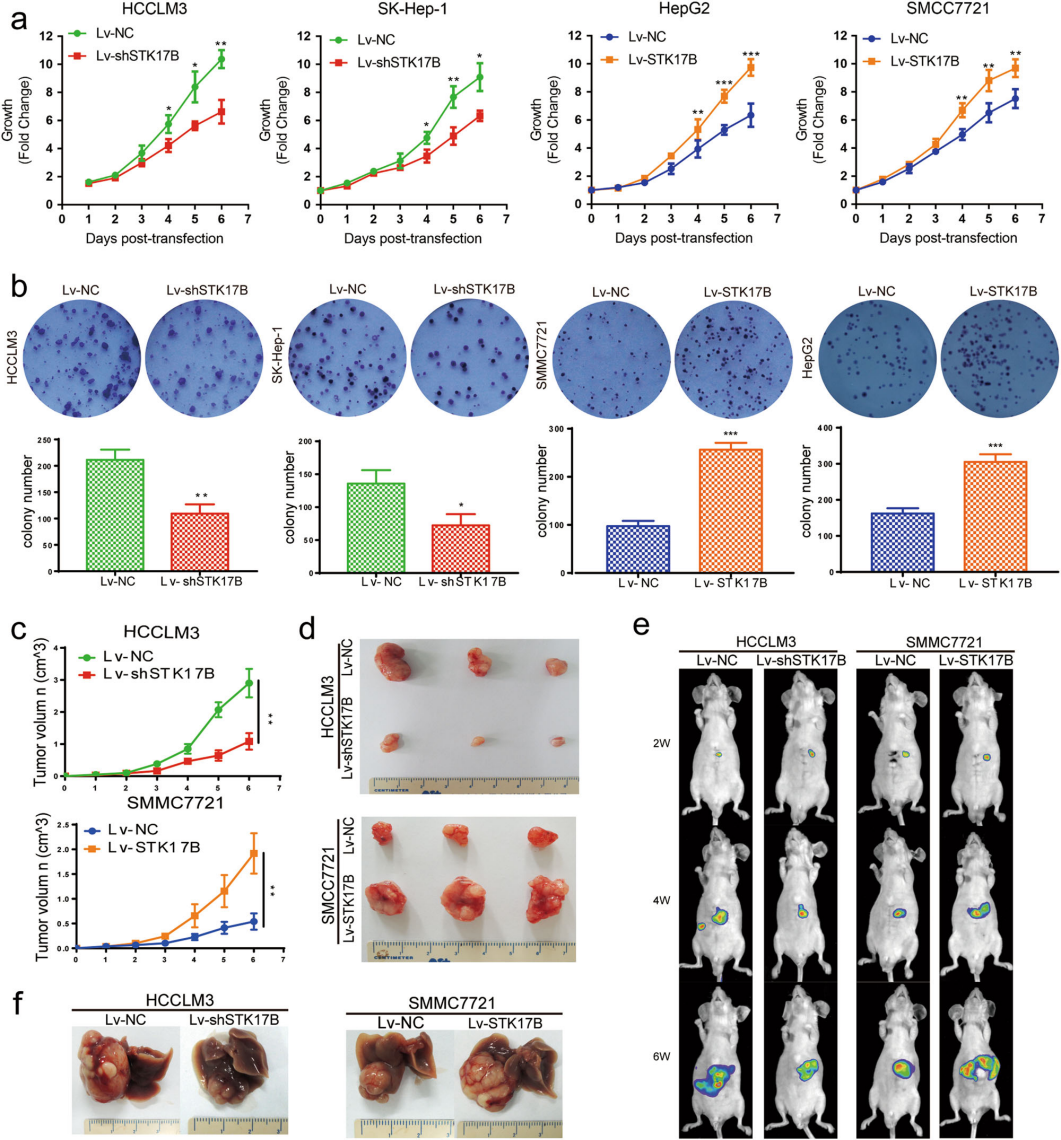
STK17B promotes proliferation and tumorigenesis both in vitro and in vivo. **a** Proliferation rate was analyzed by CCK-8 assay in indicated cell lines. **b** Representative images of colony formation were displayed in upper panel and statistical analysis were listed in lower panel. Lv-STK17B group formed lager and more colony foci, while smaller and less colony foci were found in Lv-shSTK17B group. **c** Growth curve of subcutaneous xenograft tumor revealed that STK17B promoted tumorigenesis in vivo, while silence of STK17B inhibited it. **d** Representative images of subcutaneous xenograft. **e**, **f** Orthotopic model was established. Representative images were captured by bioluminescence imaging and Nikon camera. Experiments were done three times and data are presented as mean ± SD. *P < 0.05; **P < 0.01; ***P < 0.001

To determine the role of STK17B in HCC cell proliferation in vitro, we performed STK17B gain- and loss-of-function studies. Through lentivirus transfection, STK17B was effectively overexpressed in HepG2 and SMMC7721 cells and silenced in HCCLM3 and SK-Hep-1 cells (Supplementary Figure 1a and b). After the transfection, CCK-8 and colony-formation assays were performed to investigate cell proliferation. STK17B silencing suppressed the proliferation rate, whereas STK17B overexpression increased it (Fig. 2a). In the colony-formation assay, STK17B silencing resulted in smaller and fewer colonies relative to control, while STK17B overexpression led to an increase in both colony size and number (Fig. 2b). We also detected the potential influence of STK17B on cell cycle. Interestingly, we found that silence of STK17B caused cell cycle arrest at G1 phase, whereas overexpressing STK17B promoted cell cycle progression (Supplementary Figure 1c). Furthermore, result of western blotting indicated that overexpressing STK17B increased the expression of cylcin D1 and CDK4, while silence of STK17B decreased these proteins (Supplementary Figure 1d).

To access the role of STK17B in tumorigenesis in vivo, we established a subcutaneous xenograft mouse model. Tumor growth rate and tumor volume were markedly decreased in the Lv-shSTK17B group and drastically increased in the Lv-STK17B group when compared with the Lv-NC group (Fig. 2c, d). Moreover, these conclusions were also supported by the results we obtained from orthotopic model (Fig. 2e, f). Furthermore, the results of IHC analyses showed that the subcutaneous tumor tissues from the Lv-STK17B group displayed a higher level of Ki-67 relative to control, whereas the tissues from the Lv-shSTK17B group exhibited a lower Ki-67 level (Supplementary Figure 2a and b). Taken together, these results indicated that STK17B can promote HCC cell proliferation in vitro and tumorigenesis and in vivo.

### STK17B promotes HCC cell migration and invasion in vitro and in vivo

**Fig. 3:**
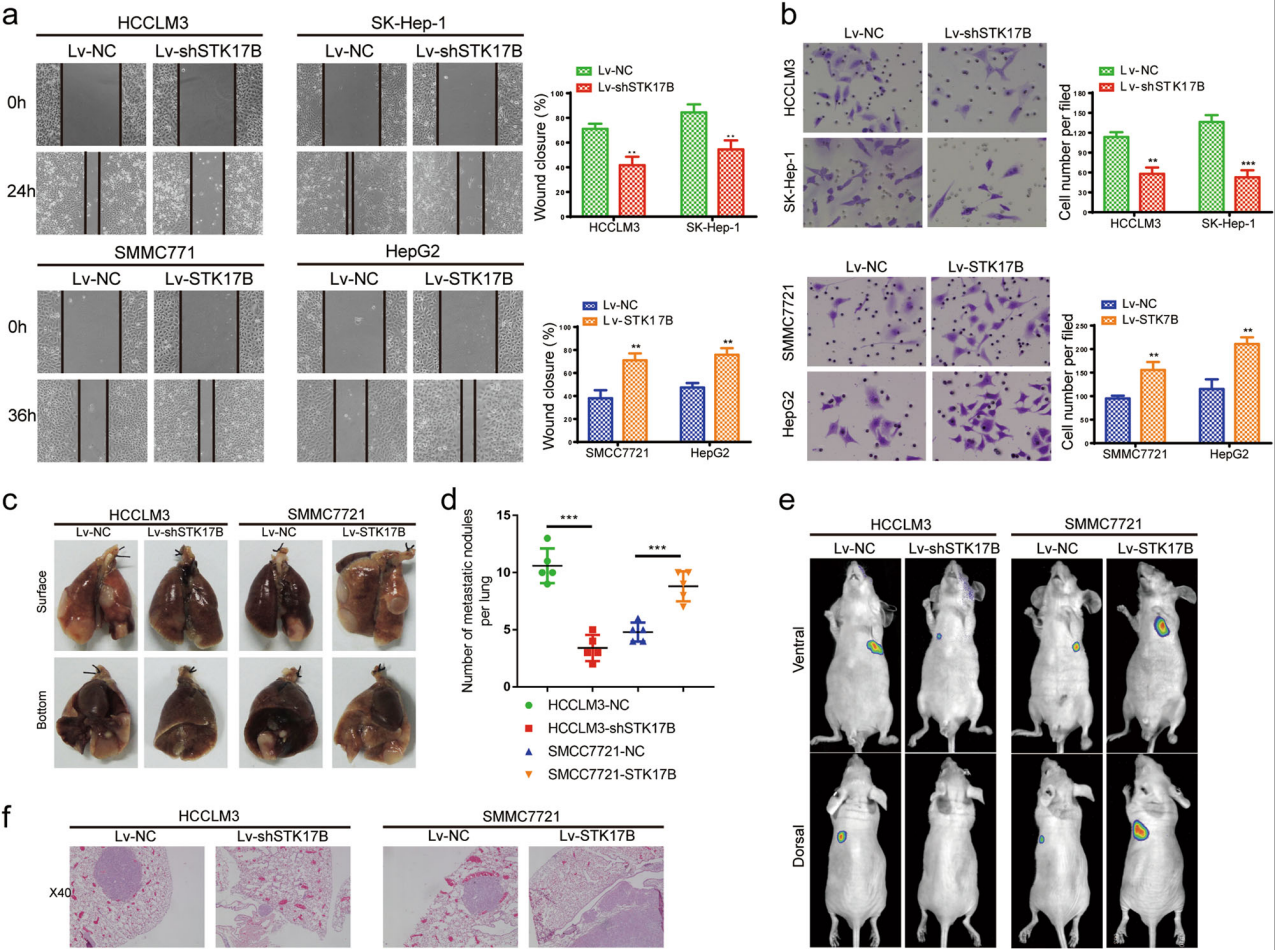
STK17B promotes invasion and metastasis both in vitro and in vivo. **a** Overexpressing STK17B promoted HCC cells migration, whereas silencing STK17B inhibited migration. Representative images were captured at 0, 24, and 36 h. **b** Representative images and corresponding statistical analysis of transwell invasion assay. STK17B overexpression promoted HCC cells invasion, while STK17B silence inhibited invasion. **c** Representative images of lung metastasis model. STK17B overexpression formed more and larger metastasis nodules, while mice in STK17B silence group burdened less and smaller metastasis nodules (*n* = 5 per group). **d** Number of lung metastasis nodule in each group. **e** Representative images of lung metastasis model were captured by bioluminescence imaging. **f** H&E staining were carried out by using lung metastasis sample. The data are expressed as mean ± SD of three independent experiments. **P < 0.01; ***P < 0.001

Clinicopathological analysis revealed that STK17B was related to TNM stage and venous invasion, which implied a potential function of STK17B in tumor migration and invasion. The results of wound-healing assays showed that when STK17B was silenced, HCC cell-migration ability was suppressed, but that the ability was enhanced when STK17B was overexpressed (Fig. 3a). Furthermore, the results of Transwell migration and invasion assays confirmed that STK17B silencing inhibited and STK17B overexpression promoted HCC cell motility and invasiveness (Fig. 3b; Supplementary Figure 3a and b).

To investigate the role of STK17B in metastasis in vivo, we constructed a nude mouse lung metastatic model by injecting stably transfected cell lines (Lv-NC, Lv-STK17B, and Lv-shSTK17B) through the tail vein. The results indicated that the number and the size of metastatic tumor nodules were decreased in mice injected with Lv-shSTK17B cells relative to control (Lv-NC), but these were increased in mice injected with Lv-STK17B (Fig. 3c, d). Moreover, the same results were confirmed by histological analysis (H&E staining) and fluorescence imaging (Fig. 3e, f). Therefore, these data provided evidence that STK17B was involved in promoting HCC cell metastasis in vitro and in vivo.

### STK17B induces EMT process in HCC

**Fig. 4:**
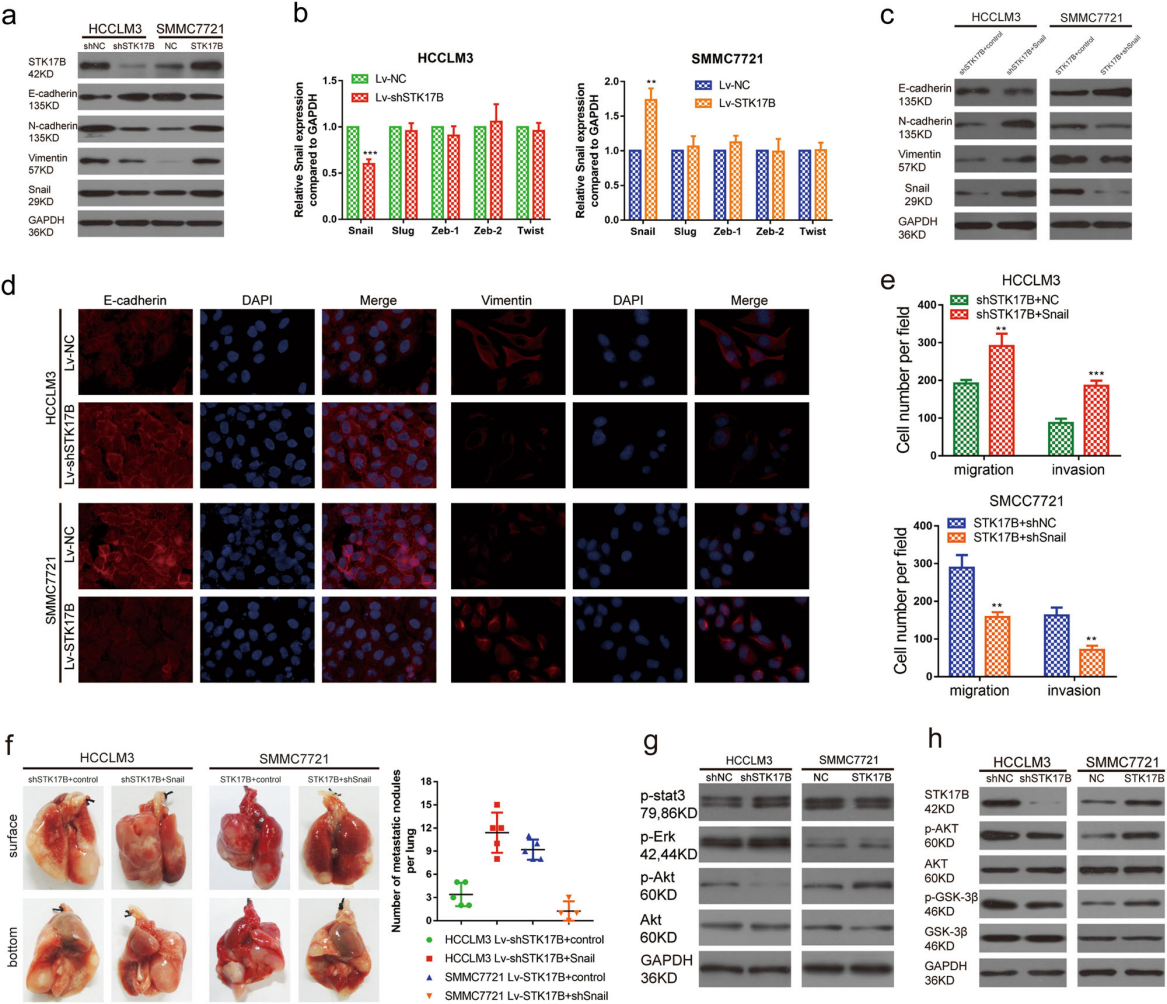
STK17B induces EMT process via activating AKT/GSK-3β/Snail pathway. **a** The expression of STK17B, E-cadherin, N-cadherin, Vimentin, and Snail was measured by western blotting. GAPDH was used as internal control. **b** mRNA expression of Snail, Slug, Zeb-1, Zeb-2, and Twist was evaluated after overexpressing or silencing STK17B. **c** Snail knockdown significantly decreased STK17B-induced EMT, while Snail overexpression enhanced it. **d** Immunofluorescence analysis of E-cadherin and vimentin expression in SMMC7721 and HCCLM3 cells. **e** Snail knockdown significantly decreased STK17B-enhanced migration and invasion, while Snail overexpression promoted shSTK17B-inhibited migration and invasion. **f** Representative images of lung metastasis nodules (left panel). Number of the metastasis nodules per lung (right panel). **g** Expression of certain key molecule in several signaling pathway was analyzed by western blotting. **h** Association between STK17B and AKT/GSK-3β/Snail signal was accessed by western blotting. All data are expressed as mean ± SD of three separate experiments. **P < 0.01; ***P < 0.001

EMT has a critical role in tumor metastasis, therefore, we sought to ascertain whether a relationship exists between STK17B and EMT. Western blotting analysis revealed that STK17B silencing led to an upregulation of the epithelial marker E-cadherin and a downregulation of the mesenchymal markers vimentin and N-cadherin relative to control (Fig. 4a). By contrast, E-cadherin expression was suppressed and vimentin and N-cadherin expression was enhanced when STK17B was overexpressed (Fig. 4a). These results were also confirmed through immunofluorescence (IF) analysis (Fig. 4d) and IHC analysis (Supplementary Figure 4a). In addition, morphological characteristic was also changed when overexpressing or silencing STK17B. Overexpression of STK17B-induced mesenchymal feature in SMMC7721, while silence of STK17B-induced epithelial feature in HCCLM3 (Supplementary Figure 4b). For the reason that Snail, Slug, Zeb-1, Zeb-2, and Twist act as key transcriptional regulators in the EMT program, we investigated the relationship between STK17B and these transcription factors. Snail mRNA expression was decreased and increased after STK17B silencing and overexpression, respectively, but the other transcription factors were not markedly altered (Fig. 4b), and this result was further confirmed by western blotting (Fig. 4a). Furthermore, we also found that STK17B overexpression-induced EMT can be attenuated by knockdown of Snail, whereas Snail overexpression can restore the EMT process inhibited by STK17B silence, as determined by measuring the expression of E-cadherin, N-cadherin, and Vimentin by western blotting (Fig. 4c).

To determine the role of Snail in STK17B-mediated migration and invasion in HCC, SMMC7721 cells transfected with Lv-STK17B plus Lv-shSnail and HCCLM3 cells transfected with Lv-shSTK17B plus Lv-Snail were used to perform further experiments. Snai1 knockdown significantly reduced STK17B-enhanced cell migration and invasion as confirmed by transwell assay, however, Snail overexpression rescued the motility and invasiveness inhibited by STK17B silence (Fig. 4e). Then, lung metastasis also indicated that more and larger lung metastasis nodules was found in mice injected with SMMC7721-STK17B-control cells than mice injected with SMMC7721-STK17B-shSnail cells, and mice in HCCLM3-shSTK17B-snail group burdened more and larger metastasis nodules than mice in HCCLM3-shSTK17B-control group (Fig. 4f). Therefore, these data indicated that Snail is critical for STK17B-induced EMT process and metastasis in HCC.

### AKT/GSK-3β/Snail pathway is involved in STK17B-induced EMT in HCC cells

Next, we examined certain key molecules in several signaling pathways in order to identify the pathway through which STK17B modulates EMT (Fig. 4g). Western blotting results showed that only the level of p-AKT was decreased and increased after STK17B silencing and overexpression, respectively, but that total AKT was unaffected (Fig. 4g). Several other studies have also revealed that the AKT/GSK-3β/Snail pathway is involved in EMT^18,19,21^. And we have obtained evidence that STK17B promotes HCC metastasis by inducing EMT through Snail. Therefore, we hypothesized that the STK17B is potentially related to the AKT/GSK-3β/Snail pathway, and we performed western blotting to test our hypothesis. We found that STK17B silencing resulted in downregulation of p-AKT, p-GSK-3β, and Snail, but did not alter total AKT and GSK-3β levels, on the contrary, STK17B overexpression increased the level of p-AKT, p-GSK-3β, and Snail (Fig. 4h). These results demonstrated that STK17B promoted EMT by activating the AKT/GSK-3β/Snail pathway.

### STK17B is a downstream target of miR-455-3p

**Fig. 5:**
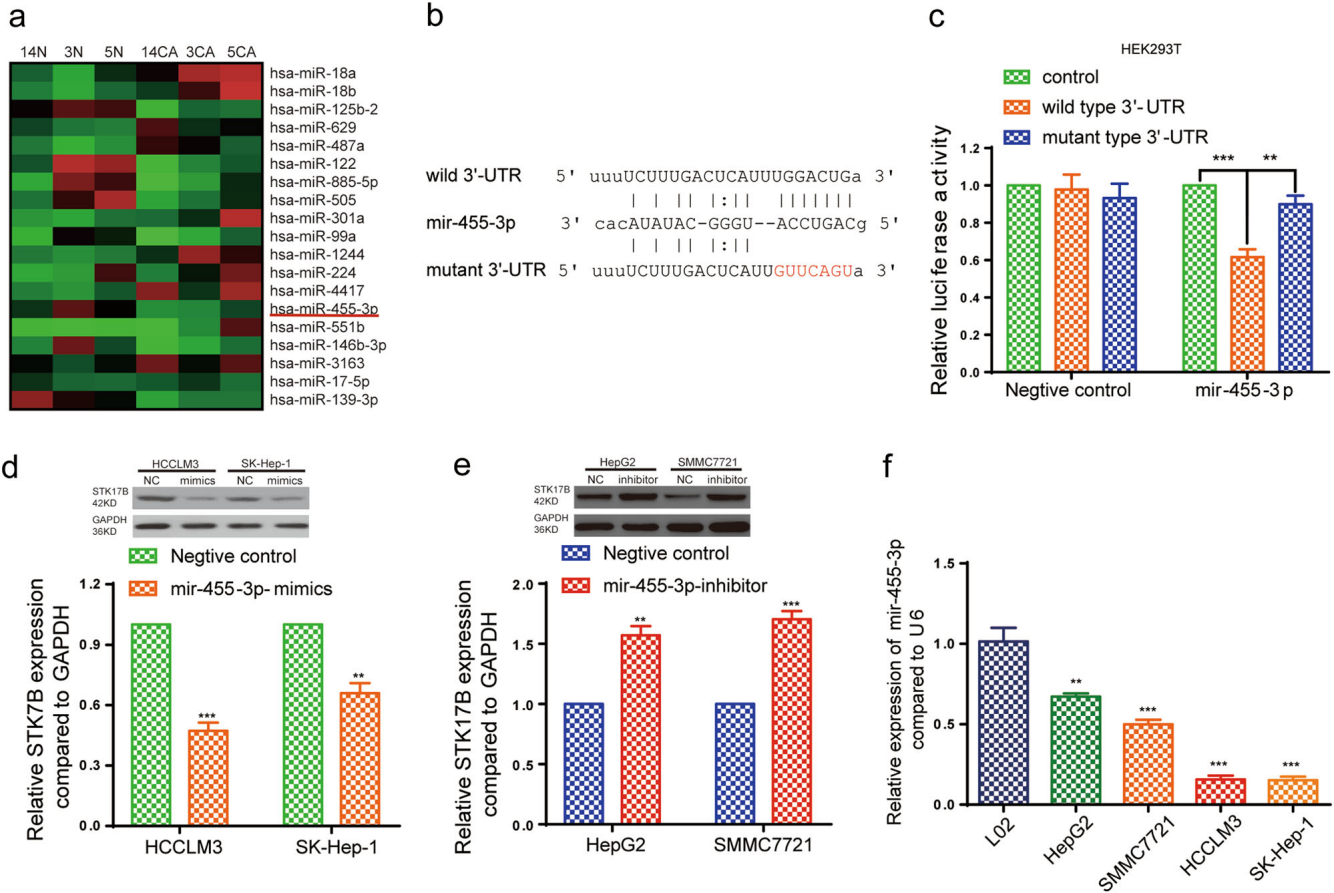
STK17B is a direct downstream target of miR-455-3p. **a** The heat map of some differentially expressed genes under gene chips analysis. **b** Binding site of miR-455-3p in wild type 3′-UTR of STK17B and the corresponding mutant type. **c** Result of luciferase reporter assay. Luciferase activity of HEK293T transfected with wild type 3′-UTR was significantly suppressed by miR-455-3p overexpression. **d**, **e** Expression of STK17B was decreased or promoted on mRNA and protein level under the condition of miR-455-3p overexpression or silencing. **f** Relative expression of miR-455-3p in normal liver cell line L02 and HCC cell lines. All data are expressed as means ± SD of three separate experiments. **P < 0.01; ***P < 0.001

More than one-third of all human genes are estimated to be regulated by miRNAs, which have essential roles in diverse biological processes as well as tumor progression ^22–27^. Therefore, by using publicly available databases, we identified several miRNAs that might function as upstream regulators of STK17B (Supplementary Figure 5a). Among these candidates, we focused on miR-455-3p, because qRT-PCR results indicated its downregulation in HCC but not other candidates (Supplementary Figure 5b) and our previous miRNA microarray analysis (using tissues from patients presenting metastasis) indicated its marked downregulation in HCC (Fig. 5a). We then designed wild and mutant 3ʹ-UTR of STK17B to perform luciferase-reporter assay (Fig. 5b), and the results confirmed that the luciferase activity was significantly inhibited by miR-455-3p overexpression in wild 3′-UTR group, but no significant alteration was found in mutant 3′-UTR group (Fig. 5c). This result indicated that STK17B is a direct target of miR-455-3p. Furthermore, both qRT-PCR and western blotting analyses revealed a negative correlation between STK17B and miR-455-3p levels (Fig. 5d, e). The qRT-PCR results indicated that miR-455-3p was downregulated in HCC tissues (Supplementary Figure 5b) and HCC cell lines (Fig. 5f). Moreover, clinicopathological analysis indicated that downregulation of miR-455-3p predicted poor clinical outcome, including venous invasion (*P* = 0.003) and TNM stage (*P* = 0.005) (Supplementary table 1). Therefore, our data suggested that STK17B is negatively regulated by miR-455-3p, which is downregulated in HCC.

### miR-455-3p inhibits HCC metastasis and EMT

**Fig. 6:**
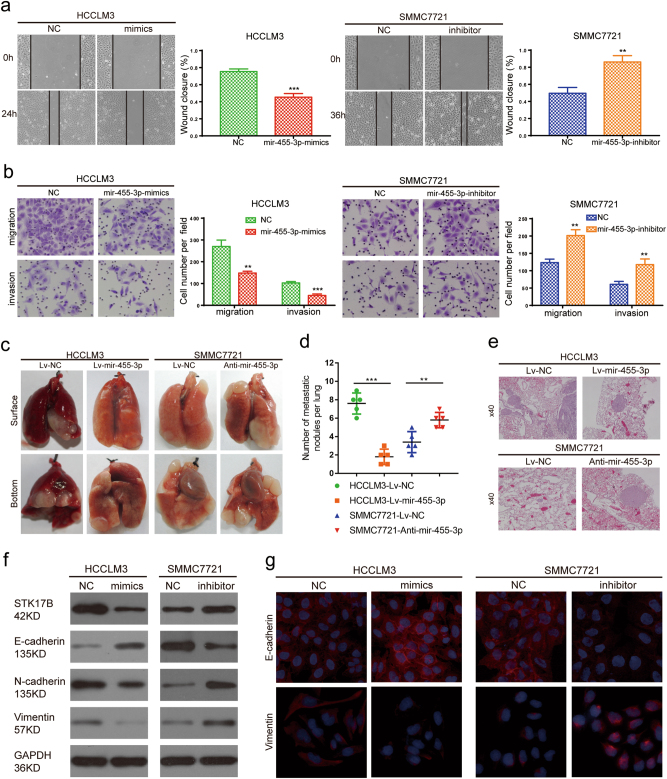
miR-455-3p inhibits invasion and metastasis both in vitro and in vivo. **a** Representative images of wound-healing assay and corresponding statistical analysis. Overexpression of miR-455-3p markedly inhibited cell motility, while knockdown of miR-455-3p promoted cell motility. **b** Results of transwell assay indicated that invasion capacity can be suppressed by miR-455-3p overexpression and enhanced by miR-455-3p knockdown. **c** Representative images of lung metastasis were captured by Nikon camera. MiR-455-3p significantly inhibited lung metastasis. **d** Number of metastatic lung nodules in each group. **e** Representative images of H&E. H&E staining were carried out using lung metastasis sample. **f** The expression of E-cadherin, N-cadherin, and Vimentin was accessed by western blotting after overexpressing of silencing miR-455-3p. **g** Immunofluorescence analysis of E-cadherin and vimentin expression. The results are presented as mean ± SD from three independent experiments. **P < 0.01; ***P < 0.001

We found that the effect of miR-455-3p on metastasis was opposite to that of STK17B in HCC. When miR-455-3p was overexpressed, HCC cell motility and invasiveness were inhibited, as determined through wound-healing and Transwell assays, whereas these abilities were enhanced when miR-455-3p was inhibited (Fig. 6a, b). Moreover, in vivo, miR-455-3p overexpression markedly decreased the incidence of lung metastasis of HCC cells relative to control, while that was drastically enhanced following miR-455-3p inhibition (Fig. 6c–e). The results of western blotting suggested that miR-455-3p can inhibit the EMT process: miR-455-3p overexpression elevated E-cadherin expression and downregulated N-cadherin and vimentin levels, but the opposite effect was observed following miR-455-3p inhibition (Fig. 6f). These findings were supported by the results of IF assays (Fig. 6g). Meanwhile, overexpressing miR-455-3p can induce epithelial characteristic and silencing miR-455-3p can promote mesenchymal characteristic when we detected the influence of miR-455-3p on morphology (Supplementary Figure 6a). To confirm the functional relationship between miR-455-3p and STK17B, rescue experiment was performed. We found that the miR-455-3p-induced inhibition of migration and invasion was attenuated when STK17B expression was restored (Supplementary Figure 6b–e). Collectively, our data suggested that miR-455-3p can inhibit metastasis and the EMT process through inhibiting STK17B in HCC.

### Combination of STK17B and miR-455-3p predicts poor prognosis in HCC patients

**Fig. 7:**
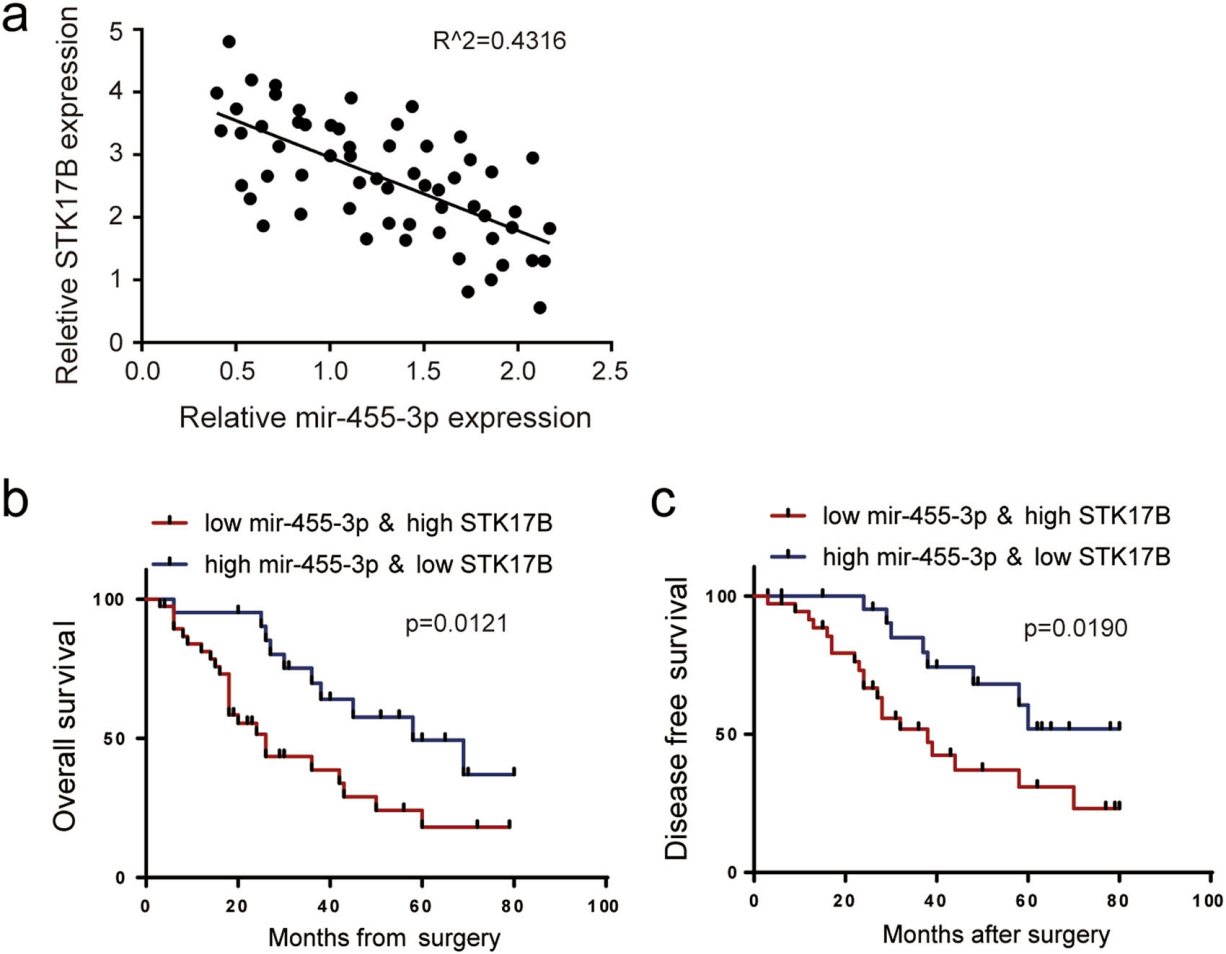
Combination of STK17B and miR-455-3p predicts prognosis with high accuracy. **a** qRT-PCR analysis indicated the inverse correlation between STK17B and miR-455-3p. **b**, **c** Kaplan–Meier analysis suggested that combination of high STK17B and low miR-455-3p predicts poor over survival rate and poor disease-free survival rate compared with low STK17B and high miR-455-3p

As noted in preceding subsections, we confirmed a reciprocal relationship between STK17B and miR-455-3p in vitro. Here, we used qRT-PCR to determine the endogenous expression levels of STK17B and miR-455-3p in HCC patient samples. In accord with our aforementioned conclusion, the results of Spearman’s correlation analysis demonstrated a strong negative correlation between STK17B and miR-455-3p in HCC tissue (Fig. 7a). Previous studies have shown that the accuracy of the prediction of patient prognosis is enhanced when several molecular makers are used together^28–30^. Here, we found that HCC patients with low level of STK17B and high level of miR-455-3p displayed higher survival rates as compared with patients expressing high level of STK17B and low level of miR-455-3p (Fig. 7b, c).

## Discussion

Efforts to elucidate the molecular mechanism underlying tumorigenicity and metastasis of HCC are urgently made, hoping to develop novel and reliable biomarkers for predicting prognosis and intervention. Previously, several studies have reported that STK17B was involved in tumor progression^10–13^, and there was also a study demonstrated its upregulation in HCC. However, the exact role of STK17B and its related mechanism still remained elusive.

In this study, we investigated the role of STK17B in HCC progression, particularly its effects on proliferation, metastasis, and invasion. We obtained evidence that STK17B was upregulated in HCC tissues, and we determined that the upregulation was associated with tumor size, TNM stage, and venous invasion by analyzing the clinicopathological features. In agreement with the clinicopathological features, our results suggested that STK17B silence suppressed tumorigenesis and caused cell cycle arrest at G1 phase in HCC. In addition, the results derived from in vitro cell migration, invasion assay, and in vivo metastasis assay confirmed that STK17B promote metastasis in HCC. Investigation of the mechanism through which STK17B promoted metastasis revealed that STK17B overexpression promoted the EMT process. We found STK17B can induce HCC cell to gain mesenchymal feature. Meanwhile, epithelial maker (E-cadherin) was suppressed while mesenchymal makers (N-cadherin and Vimentin) were increased by STK17B. To clarify the regulatory interaction between STK17B and EMT, several transcription factors which were upstream regulators of EMT and EMT-related signal pathways were measured. Finally, Snail and AKT pathway were found to be positively related to STK17B. Previous studies have revealed that AKT/GSK-3β/Snail pathway is widely accepted in modulating EMT^20,21^, therefore we hypothesized that there might be a relationship between STK17B and this pathway. Our western blotting results indicated that STK17B expression can result in elevation of phosphorylated AKT, and thereby inhibition of GSK-3β and upregulation of Snail. Subsequently, activation of this pathway modulates E-cadherin, N-cadherin, and vimentin expression and thereby induces EMT.

Various miRNAs have been demonstrated to be aberrantly expressed in malignant tumors, including HCC^31^, and almost one-third of all human genes are regulated by miRNAs. Here, we identified miR-455-3p, which has been reported to be associated with HCC^32,33^, as an upstream suppressor of STK17B by performing bioinformatics analysis and luciferase reporter assy. Accordingly, miR-455-3p attenuated the function of STK17B, and the results of qRT-PCR analysis of HCC specimens confirmed a negative correlation between miR-455-3p and STK17B expression. Clinicopathological analysis also confirmed downregulation of miR-455-3p predicts poor clinical outcomes. Moreover, we demonstrated that a combination of high STK17B expression and low miR-455-3p expression predicts a poor prognosis in HCC patients.

Taken together, the present study identified that STK17B is upregulated in HCC tissues and cell lines, and may predicts poor clinical outcomes. Moreover, STK17B might function as a regulator of HCC carcinogenesis and metastasis. Interestingly, STK17B, miR-455-3p, and the AKT/GSK-3β/Snail pathway might operate in combination in regulating the EMT process—and thereby metastasis—in HCC.

## Materials and methods

### Cell lines and cell culture

An immortalized normal liver cell line (L02) and several HCC cell lines (SMMC7721, Huh7, HCCLM3, HepG2, Sk-Hep-1, and Hep3B) were purchased from Cell Bank of Type Culture Collection of the Chinese Academy of Sciences, Shanghai Institute of Cell Biology, Chinese Academy of Sciences. All cell lines were cultured in Dulbecco’s modified Eagle’s medium (DMEM) supplemented with 10% fetal bovine serum and 1% antibiotics (100 U/ml penicillin and 100 μg/ml streptomycin) at 37 °C in a 5% CO_2_ incubator.

### Patients and clinical samples

HCC tissues and corresponding adjacent non-tumorous liver tissues (ANLTs) were collected from patients on whom hepatic resection was performed at the First Affiliated Hospital of Harbin Medical University between January 2008 and May 2013. Informed consent was obtained from all patients, and the research application for human subjects was approved by the Research Ethics Committee of the First Affiliated Hospital of Harbin Medical University. Every HCC diagnosis was based on World Health Organization criteria. Follow-up data were regularly collected from patients after hepatic resection to access the overall rate of and to monitor the cancer metastasis and recurrence. The patents’ clinicopathological data are presented in Supplementary Table 2.

### Lentivirus, oligonucleotides, and reagents

Lentiviral vectors for STK17B gene overexpression (Lv-STK17B) and downregulation (Lv-shSTK17B) were constructed by and purchased from GeneChem Corporation (Shanghai, China); the empty vector (Lv-NC) was used as a negative control. Virus for interfering Snail was also bought from GeneChem Corporation. A lentiviral vector encoding the firefly luciferase gene was constructed by and purchased from GeneChem Corporation. All other lentiviral vectors were designed by and purchased from GeneChem Technologies. Oligonucleotides for miR-455-3p overexpression (mimics) and knockdown (inhibitor), and the corresponding negative-control (NC) oligonucleotide, were purchased from RiboBio Corporation (Guangzhou, China). Information on all the primary antibodies used here is provided in Supplementary Table 3. Detailed information regarding the primers and probes used for quantitative real-time PCR (qRT-PCR) is listed in Supplementary Table 4.

### Immunohistochemical (IHC) analysis

Formalin-fixed and paraffin-embedded tissue sections were deparaffinized in xylene and rehydrated in a gradient ethanol series, after which antigen retrieval was performed in an antigen-unmasking solution (citrate-based). After blocking with normal bovine serum, the sections were incubated with primary antibodies at optimal dilutions (overnight, 4 °C) and then with biotinylated secondary antibodies (Vector Laboratories, Burlingame, CA, USA). Lastly, the sections were stained with diaminobenzidine (DAB Kit; Vector Laboratories) and counterstained with hematoxylin (Sigma, St. Louis, MO, USA) to visualize the immunoreaction product. Staining density was evaluated using an IHC method described previously^34^.

### Cell-viability and colony-formation assays

Transfected cells were seeded in 96-well plates (1 × 10^3^ cells per well) and incubated overnight at 37 °C under 5% CO_2_ to allow attachment. Cell viability at various time points was measured using Cell Counting Kit-8 (CCK-8) assays (CK04-01; Dojindo Molecular Technologies, Inc., Japan). The primary medium in the wells was replaced with 100 μl of complete medium supplemented with 10 μl of CCK-8 reagent, and after incubating at 37 °C under 5% CO_2_ for 4 h, 450-nm absorbance was measured. Six replicates were used for each experiment.

For colony-formation assays, transfected cells were seeded in 6-well plates at 300–500 cells per well and incubated at 37 °C under 5% CO2 for 14 days. Subsequently, the cells were fixed by adding 4% (w/v) paraformaldehyde into each well and incubating for 10 min, after which staining was performed with 0.5% crystal violet to visualize colonies. Images were captured using a Nikon camera.

### Wound-healing assay

Cells were seeded in 6-well plates and cultured at 37 °C under 5% CO_2_ for 24 h to ensure that the cells grew to confluence, after which a wound was scratched in each well by using a 10-μl pipette tip. Cells were washed thrice with phosphate-buffered saline (PBS) and then 2 ml of complete culture medium was added into each well. Wound closure was monitored at 0, 24, and 36 h, and representative scratch lines were photographed by using an inverted microscope equipped with a Nikon camera. Three replicated wells were established for each condition.

### Transwell assay

In vitro invasion capability was measured using 24-well BioCoat cell-culture inserts (BD Biosciences, NJ, USA) pre-coated or not pre-coated with Matrigel on the polyethylene terephthalate membrane. In the upper compartment of the Transwell chambers, we seeded 2 × 10^4^ cells suspended in 500 μl of serum-free medium, and we filled the lower compartment with complete medium. After incubation at 37 °C under 5% CO_2_ for 24–48 h, some of the cells had migrated through the porous membrane, whereas the non-migrating cells remained on the upper surface of the filter. The chamber was now immersed in methanol for 10 min to fix the cells, and then scrubbed gently to remove the non-metastatic cells. The migrated cells were stained with 0.5% crystal violet for 10 min and counted under a light microscope. Each experiment was independently performed thrice.

### Western blotting

Harvested cells were lysed in RIPA buffer supplemented with protease and phosphatase inhibitors to prepare total protein samples. After measuring protein concentrations, the samples were separated using SDS-PAGE and then electro-transferred onto polyvinylidene fluoride (PVDF) membranes (Invitrogen, Eugene, OR, USA). The PVDF membranes were blocked in 5% skim milk for 1 h at room temperature, incubated overnight at 4 °C with primary antibodies at optimal dilution, washed with PBST (PBS containing 0.1% Tween-20), and incubated with HRP-conjugated secondary antibodies. Lastly, protein expression was visualized by detecting the immunoreactive bands on the membranes by using an enhanced chemiluminescence kit (Pierce, Rockford, IL, USA).

### Real-time PCR

Total RNA was extracted from cultured cells by using an RNeasy Mini Kit (Qiagen, Valencia, CA) according to the manufacturer’s instructions, and the RNA was reverse-transcribed into cDNA by using a High Capacity Reverse Transcription Kit (Applied Biosystems, Grand Island, NY). Real-time PCR was conducted using Power SYBR Green PCR Master Mix (Life Technologies, USA), on an ABIPRISM 7900HT instrument (Applied Biosystems, Grand Island, NY), and mRNA expression levels were normalized relative to the GAPDH mRNA level. For microRNA (miRNA) analyses, RT-PCR and real-time PCR were performed using, respectively, a TaqMan® MicroRNA Reverse Transcription Kit and TaqMan® Universal Master Mix II no UNG (both from Applied Biosystems, Grand Island, NY). The miRNA expression level was normalized relative to the U6 level.

### Cell cycle analysis

Cells (4 × 10^5^) were fixed in 70% ethanol for 1 h at 4 °C. Then the cells were washed twice with PBS followed by adding 10 mg/ml RNase A. After that propidium iodide was added at a final concentration of 0.05 mg/ml, the samples were incubated at 4 °C for 30 min in a dark environment. Finally, samples were analyzed by flow cytometry (Beckman Coulter FC 500).

### Immunofluorescence (IF) assay

Cultured cells were seeded on coverslips in advance and fixed with 4% (w/v) paraformaldehyde for 10 min. Subsequently, the cells were permeabilized for 20 min in 0.1% (v/v) Triton X-100 at room temperature, incubated with primary antibodies overnight at 4 °C and then with fluorescent secondary antibodies (Invitrogen) for 1 h at room temperature, and washed thrice with PBS. Lastly, the cells counterstained with DAPI (Vector Laboratories) and then images were captured.

### Luciferase assay

Luciferase activity was accessed using a luciferase assay kit (Promega, Madison, WI, USA). Cells were seeded in 24-well plates and incubated for 24 h, and then specific plasmids were cotransfected with 1 ng of pRL-TK Renilla luciferase plasmid into the cells by using Lipofectamine 2000 (Invitrogen). At 48 h after transfection, the Dual-Luciferase Reporter Assay System (Promega) was used to measure luciferase activity according to the manufacturer’s instructions.

### Animal studies

Male BALB/c athymic nude mice (4–6 weeks old) were purchased from the experimental animal center of Shanghai Institute for Biological Sciences. The experimental protocol was reviewed and approved by the Committee on the Use of Live Animals in Teaching and Research of the Harbin Medical University, Harbin, China. All animals were housed under standard pathogen-free conditions. To establish subcutaneous xenograft tumors, 2 × 10^6^ cells suspended in 100 μl of PBS were injected into the flank of mice. Tumor size was monitored weekly by using Vernier calipers until all animal were killed at the 6th week. Tumor volume was calculated as previously reported^35^.

Subcutaneous xenograft tumors can be visualized after 1 week. Tumors were resected and diced into 1 mm^3^ cubes, which were then implanted into the left lobe of the liver in mice. Subsequently, tumor size was monitored by measuring the bioluminescence signal every week until all mice were killed for collecting tumor tissues at the 6th week.

The lung metastasis model was established to access in vivo metastasis: 3 × 10^6^ cells were injected into nude mice through the tail vein, and 6 weeks later, bioluminescence was measured using a Berthold NIGHTOWL LB983 imaging machine. After the imaging, all mice were killed, and the lung was resected for counting metastatic nodules and for hematoxylin–eosin (H&E) staining.

### Statistical analysis

Statistical analysis was performed using SPSS 16.0 software (SPSS, Chicago, IL, USA) and the GraphPad Prism software package (v. 6.01, San Diego, CA, USA). All data are presented as means ± SD. Variance between two groups was analyzed using unpaired Student’s *t* tests. *P* < 0.05 was considered statistically significant.

### Disclaimer

The funders had no role in study design, data collection and analysis, decision to publish, or preparation of the manuscript.

## Electronic supplementary material

supplementary figure 1

supplementary figure 2

supplementary figure 3

supplementary figure 4

supplementary figure 5

supplementary figure 6

Supplementary figure legendary

Supplementary tables
