# Supplementary figures and images for: STK17B promotes carcinogenesis and metastasis via AKT/GSK-3β/Snail signaling in hepatocellular carcinoma
Source: Cell Death Dis. 2018 Feb 14;9(2):236. doi: 10.1038/s41419-018-0262-1 (PMC5833726; doi:10.1038/s41419-018-0262-1)

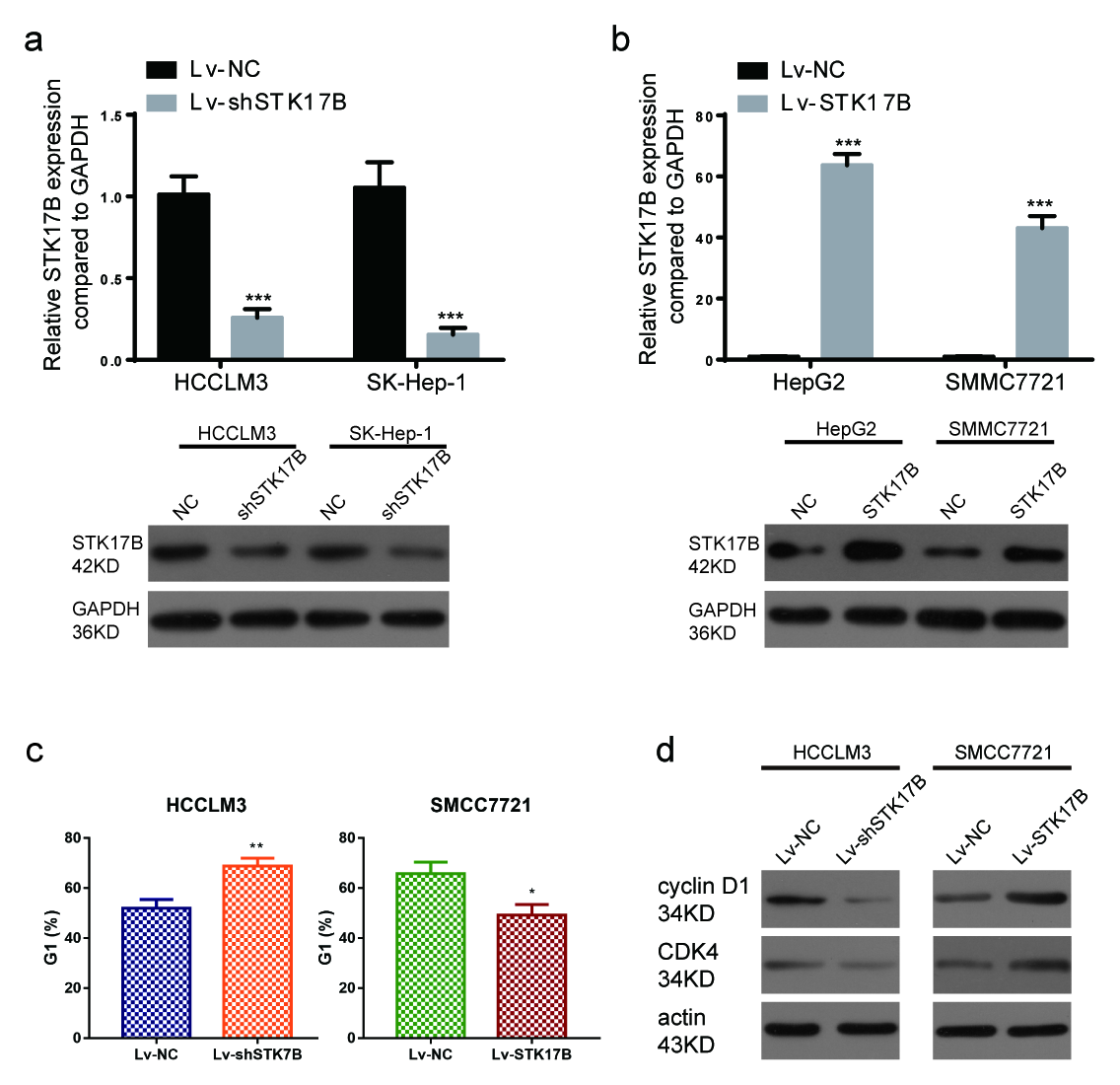

Supplement: Supplementary file 1 — supplementary figure 1 [file 41419_2018_262_MOESM1_ESM.tif]

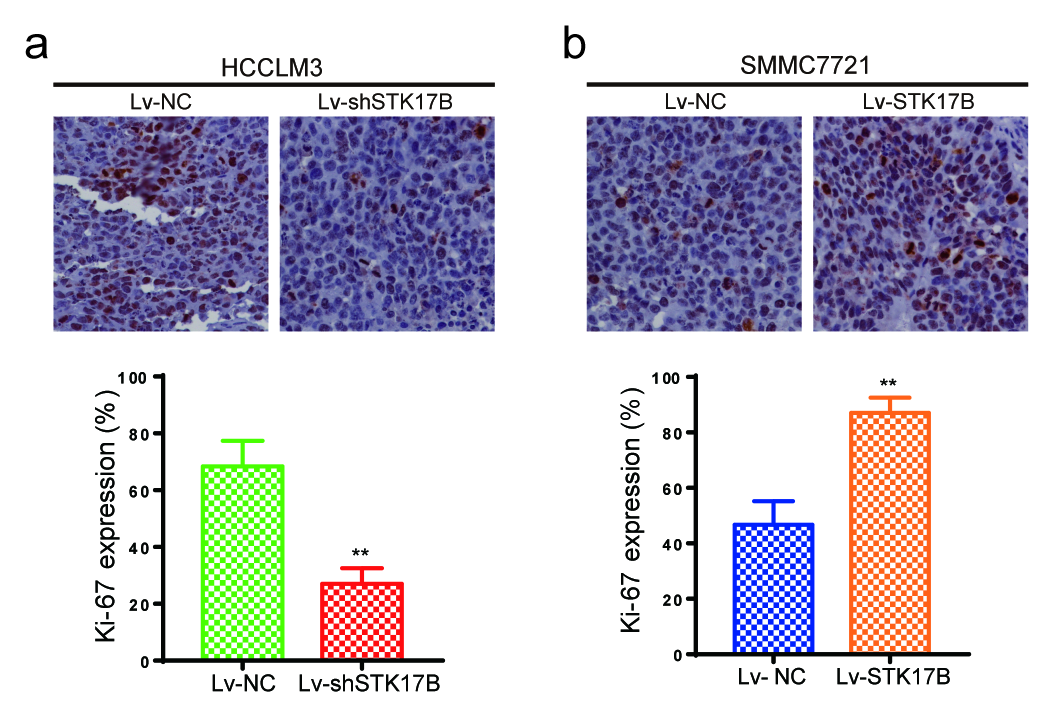

Supplement: Supplementary file 2 — supplementary figure 2 [file 41419_2018_262_MOESM2_ESM.tif]

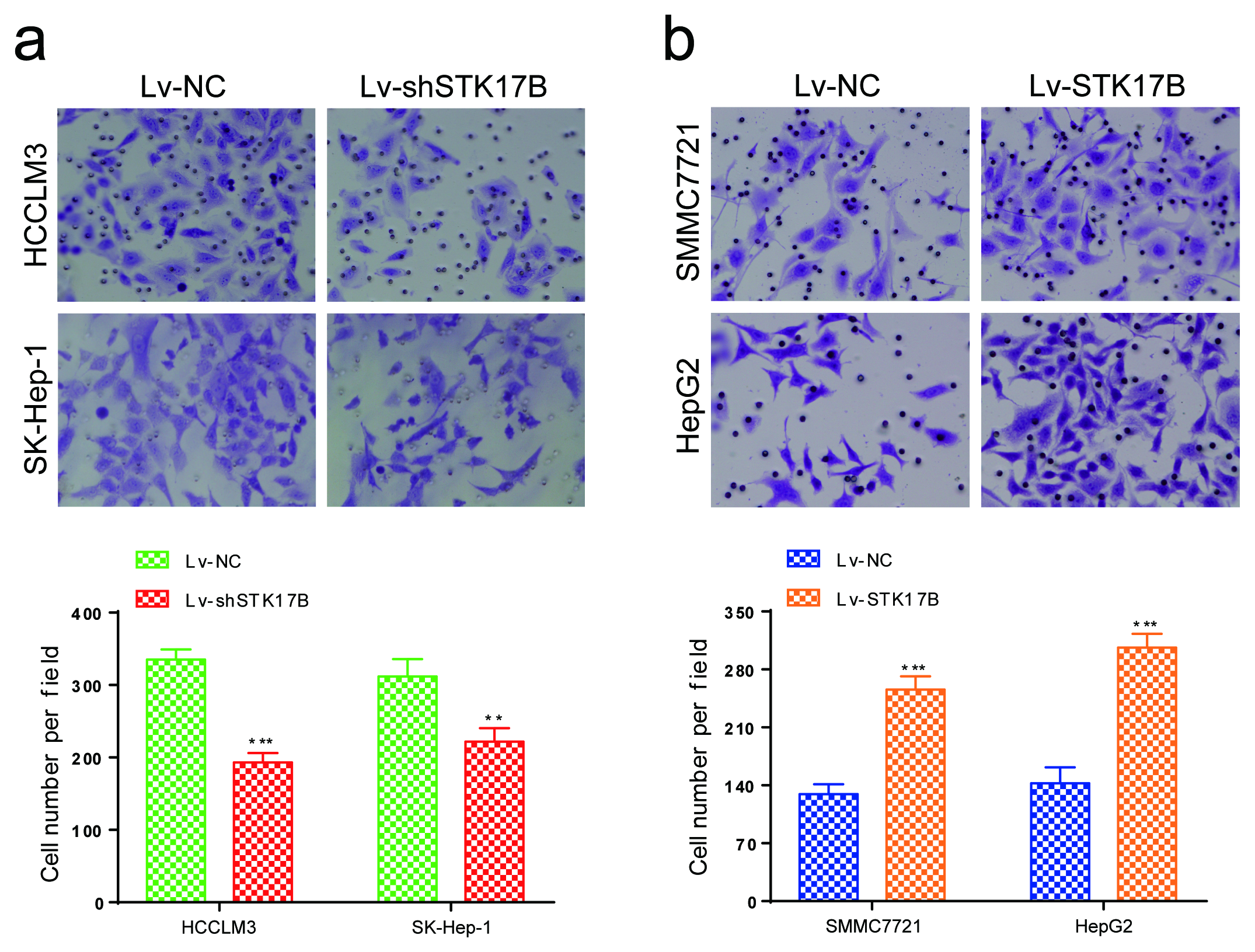

Supplement: Supplementary file 3 — supplementary figure 3 [file 41419_2018_262_MOESM3_ESM.tif]

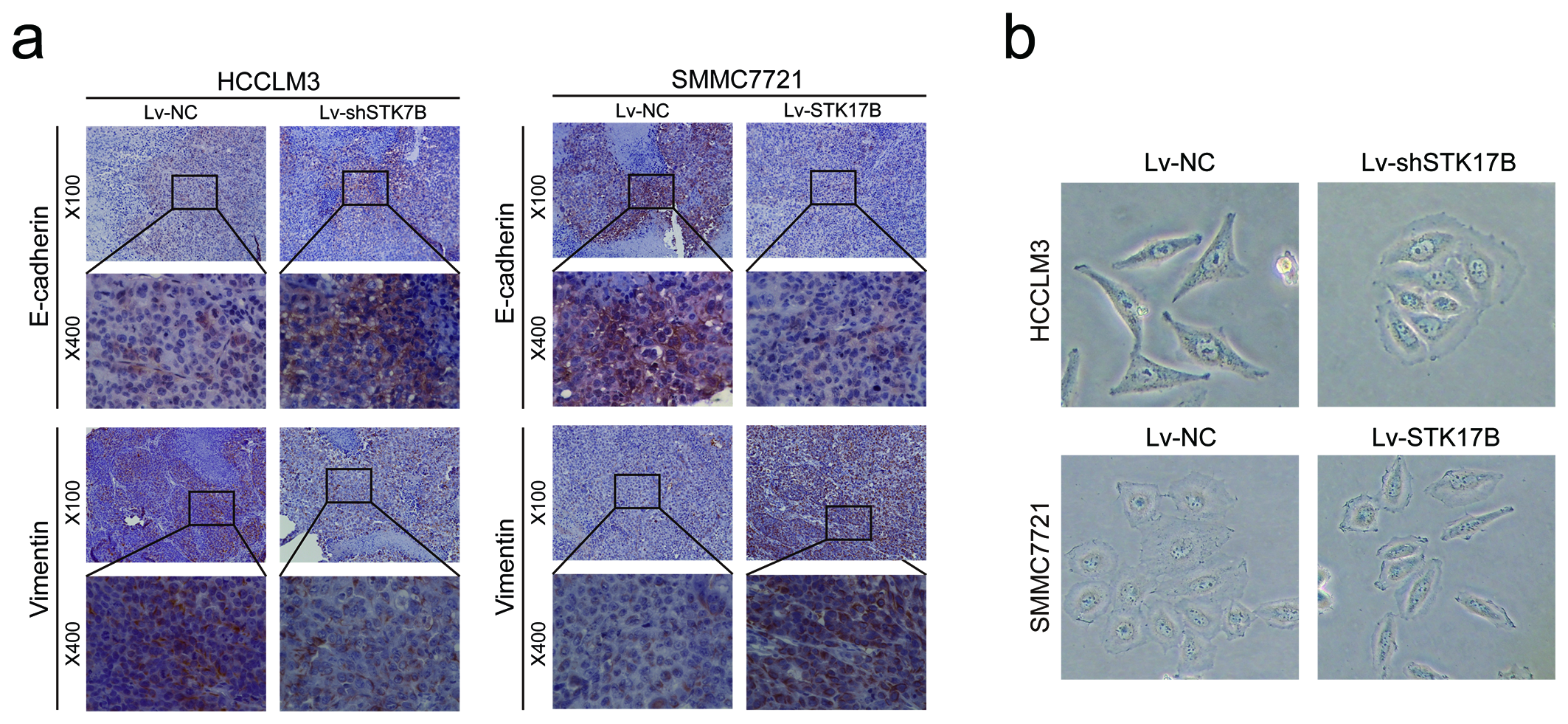

Supplement: Supplementary file 4 — supplementary figure 4 [file 41419_2018_262_MOESM4_ESM.tif]

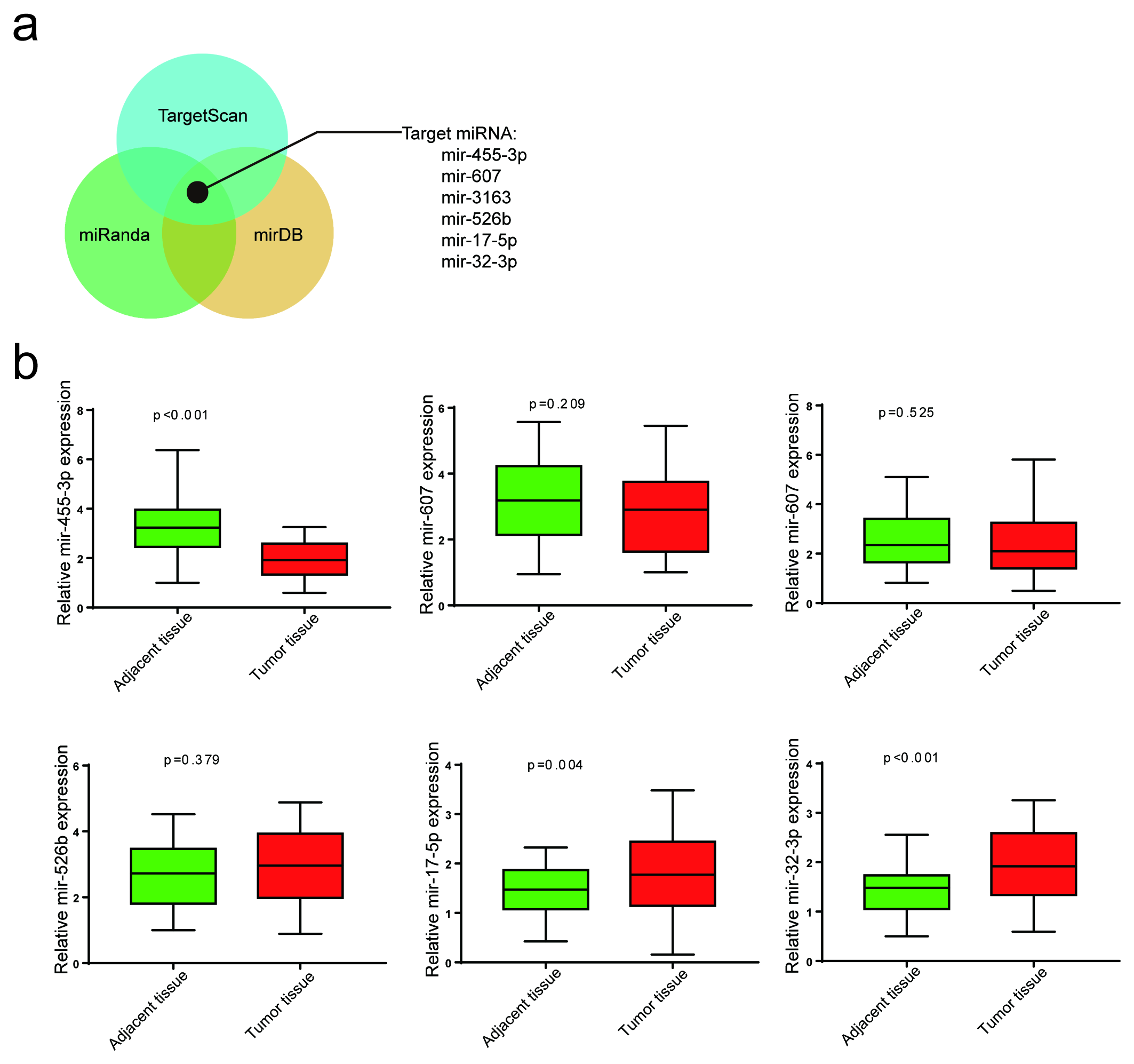

Supplement: Supplementary file 5 — supplementary figure 5 [file 41419_2018_262_MOESM5_ESM.tif]

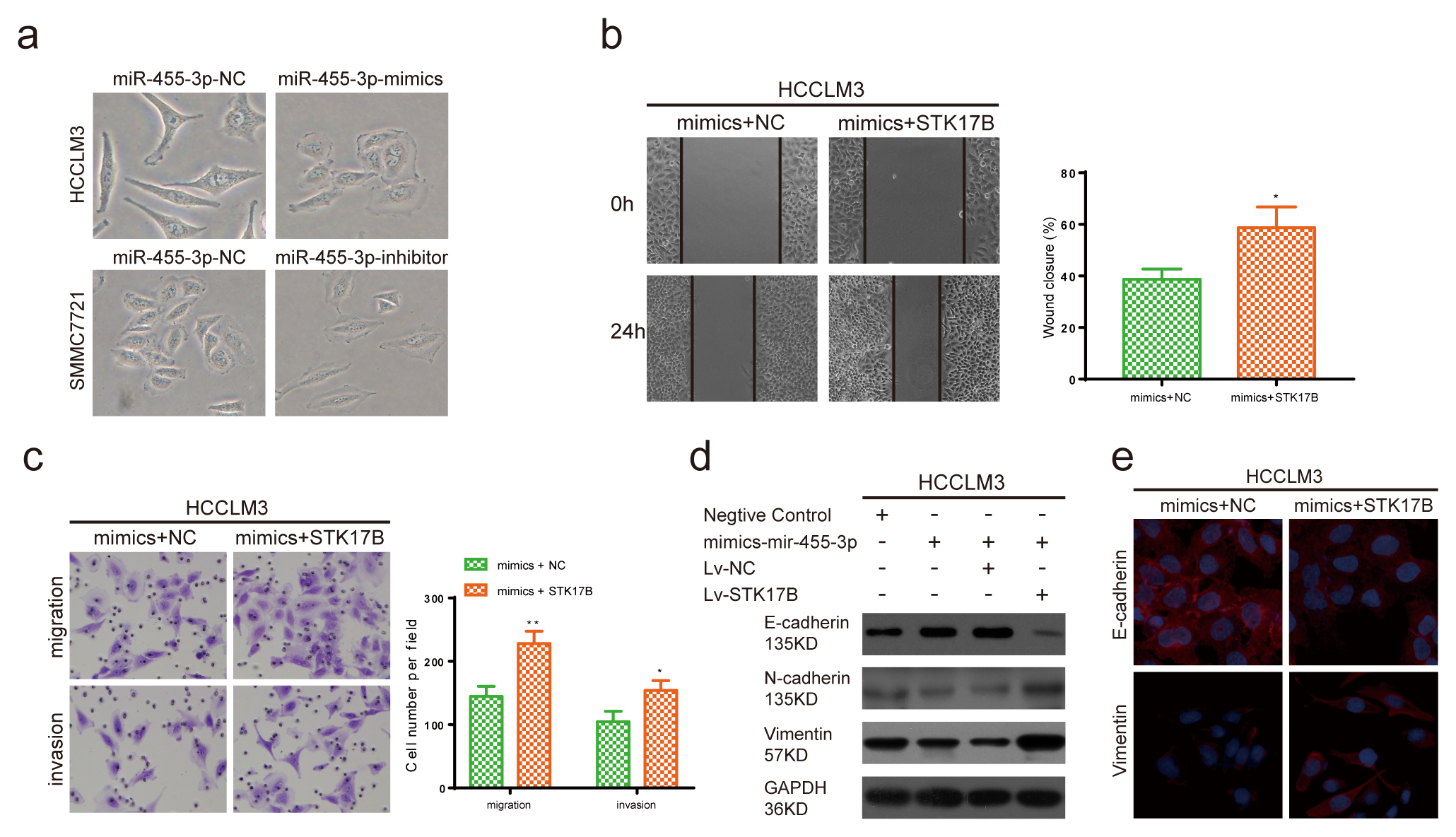

Supplement: Supplementary file 6 — supplementary figure 6 [file 41419_2018_262_MOESM6_ESM.tif]
