# Supplementary material for: STK17B promotes carcinogenesis and metastasis via AKT/GSK-3β/Snail signaling in hepatocellular carcinoma
Source: Cell Death Dis. 2018 Feb 14;9(2):236. doi: 10.1038/s41419-018-0262-1 (PMC5833726; doi:10.1038/s41419-018-0262-1)
Supplement: Supplementary file 7 — Supplementary figure legendary [file 41419_2018_262_MOESM7_ESM.docx]

**Supplementary figure legends**

**Figure 1** Expression of STK17B in indicated HCC cells was measured by qRT-PCR on mRNA level. **(a)** STK17B was efficiently silenced in HCCLM3 and SK-Hep-1 as determined by qRT-PCR and western blot. **(b)** STK17B was efficiently overexpressed in HepG2 and SMCC7721 on mRNA and protein level. **(c)** The cell cycle distribution of HCCLM3 and SK-Hep-1 (NC and KD) cells were analyzed. Knockdown of STK17B cause cell cycle arrest at G1 phase. **(d)** The expression level of cyclin D1 and CDK4 was measured by western blotting. The results are presented as mean ± SD from three independent experiments. ****P* < 0.001.

**Figure 2** IHC analysis of Ki-67 expression level. **(a-b)** Expression of Ki-67 was increased and decreased on the condition of STK17B overexpression and silence respectively. The results are presented as mean ± SD from three independent experiments. ***P* < 0.01.

**Figure 3** Representative images transwell migration and corresponding statistical analysis. **(a)** STK17B silence inhibited HCC cells migration. **(b)** STK17B overexpression promoted HCC cells migration. The results are presented as mean ± SD from three independent experiments. ***P* < 0.01; ****P* < 0.001.

**Figure 4 (a)** Representative images of IHC staining. Expression of E-cadherin and Vimentin was evaluated by IHC staining using subcutaneous xenograft tumor tissues. **(b)** Effect of STK17B overexpression or inhibition on cell morphology evaluated by phase-contrast microscopy.

**Figure 5** Candidate microRNAs and their expression in HCC tissue and ANLTs **(a)** Candidate miRNAs were screened by using public available database. **(b)** The expression level of the candidate miRNAs was analyzed by qRT-PCR. The results are presented as mean ± SD from three independent experiments.

**Figure 6** The inhibited migration and invasion induced by mir-455-3p can be attenuated by STK17B restoration. **(a)** Effect of miR-455-3p overexpression or inhibition on cell morphology evaluated by phase-contrast microscopy. **(b)** Representative images of wound healing and matched statistical analysis. **(c)** Representative images of transwell assay and corresponding statistical analysis. **(d)** The expression level of E-cadherin, N-cadherin and Vimentin was measured by western blotting. **(e)** The expression level of E-cadherin and Vimentin was measured by IF assay. The results are presented as mean ± SD from three independent experiments. **P* < 0.05; ***P* < 0.01.
