# Supplementary material for: STK17B promotes carcinogenesis and metastasis via AKT/GSK-3β/Snail signaling in hepatocellular carcinoma
Source: Cell Death Dis. 2018 Feb 14;9(2):236. doi: 10.1038/s41419-018-0262-1 (PMC5833726; doi:10.1038/s41419-018-0262-1)
Supplement: Supplementary file 8 — Supplementary tables [file 41419_2018_262_MOESM8_ESM.docx]

**Table 1. Correlations of miR-455-3p With Clinicopathological Features of HCC (n=60)**

|  | | mir-455-3p | |  |
| --- | --- | --- | --- | --- |
| Clinicopathologic Variable | n | Low level | High level | *P* value |
| Gender |  |  |  |  |
| Female | 23 | 12 | 11 | 0.163 |
| Male | 37 | 27 | 10 |  |
| Age(years) |  |  |  |  |
| ≤60 | 31 | 19 | 12 | 0.595 |
| >60 | 29 | 20 | 9 |  |
| AFP(ng/mL) |  |  |  |  |
| ≤20 | 25 | 14 | 11 | 0.276 |
| >20 | 35 | 25 | 10 |  |
| HBsAg |  |  |  |  |
| Negative | 20 | 11 | 9 | 0.268 |
| Positive | 40 | 28 | 12 |  |
| Liver cirrhosis |  |  |  |  |
| Absence | 28 | 22 | 6 | 0.058 |
| Presence | 32 | 17 | 15 |  |
| Tumor size(cm) |  |  |  |  |
| ≤5 | 22 | 13 | 9 | 0.577 |
| >5 | 38 | 26 | 12 |  |
| Venous invasion |  |  |  |  |
| Absence | 36 | 29 | 7 | **0.003** |
| Presence | 24 | 10 | 14 |  |
| TNM stage |  |  |  |  |
| I–II | 22 | 9 | 13 | **0.005** |
| III–IV | 38 | 30 | 8 |  |
| Liver function |  |  |  |  |
| Child-Pugh A | 30 | 16 | 14 | 0.103 |
| Child-Pugh B | 30 | 23 | 7 |  |

Abbreviations: AFP, alpha-fetoprotein; HBsAg, hepatitis B surface antigen; TNM, tumor node metastasis.

|  | |  | STK17B | |  |
| --- | --- | --- | --- | --- | --- |
| Clinicopathologic Variable | n | | Low level | High level | *P* value |
| Gender |  | |  |  |  |
| Female | 26 | | 14 | 12 | 0.068 |
| Male | 34 | | 10 | 24 |  |
| Age(years) |  | |  |  |  |
| ≤60 | 30 | | 11 | 19 | 0.793 |
| >60 | 30 | | 13 | 17 |  |
| AFP(ng/mL) |  | |  |  |  |
| ≤20 | 27 | | 8 | 19 | 0.188 |
| >20 | 33 | | 16 | 17 |  |
| HBsAg |  | |  |  |  |
| Negtive | 27 | | 7 | 20 | 0.064 |
| Positive | 33 | | 17 | 16 |  |
| Liver cirrhosis |  | |  |  |  |
| Absence | 27 | | 12 | 15 | 0.601 |
| Presence | 33 | | 12 | 21 |  |
| Tumor size(cm) |  | |  |  |  |
| ≤5 | 18 | | 11 | 7 | **0.044** |
| >5 | 42 | | 13 | 29 |  |
| Venous invasion |  | |  |  |  |
| Absence | 24 | | 14 | 10 | **0.031** |
| Presence | 36 | | 10 | 26 |  |
| TNM stage |  | |  |  |  |
| I–II | 19 | | 13 | 6 | **0.004** |
| III–IV | 41 | | 11 | 30 |  |
| Liver function |  | |  |  |  |
| Child-Pugh A | 31 | | 15 | 16 | 0.197 |
| Child-Pugh B | 29 | | 9 | 20 |  |

**Table 2. Correlations between STK17B and Clinicopathological Features of HCC patients (n=60)**

Abbreviations: AFP, alpha-fetoprotein; HBsAg, hepatitis B surface antigen; TNM, tumor node metastasis.

**Table 3. Primary antibodies for WB and IHC**

| Protein | Concentration for WB | Concentration for IHC | Specificity | Company |
| --- | --- | --- | --- | --- |
|  |  |  |  |  |
| STK17B | 1：1000 | 1：100 | Rabbit | Abcam& Cell Signaling |
| Ki-67 | - | 1：100 | Rabbit | Abcam |
| E-cadherin | 1：1000 | 1：100 | Mouse | Abcam |
| Vimentin | 1：4000 | 1：100 | Mouse | Abcam |
| β-actin | 1：4000 | - | Mouse | Sigma |
| GAPDH | 1：4000 | - | Mouse | Cell Signaling |
| GSK-3β | 1：1000 | - | Rabbit | Abcam |
| p-GSK-3β^Ser9^ | 1：1000 | - | Rabbit | Abcam |
| AKT | 1：1000 | - | Rabbit | Cell Signaling |
| p-AKT^Ser473^ | 1：1000 | - | Rabbit | Cell Signaling |
| ERK | 1：1000 | - | Rabbit | Cell Signaling |
| p-ERK^Thr202/Thr204^ | 1：1000 | - | Rabbit | Cell Signaling |
| Snail | 1：1000 | - | Rabbit | Cell Signaling |
| Cyclin D1 | 1：1000 | - | Rabbit | Abcam |
| CDK4 | 1：1000 | - | Rabbit | Abcam |

**Table 4. Primers used in the study**

| **Primers** |  | sequences (5'-3') |
| --- | --- | --- |
| STK17B | sense: | 5'-ATCAACTTGACCATTCTGGCT-3' |
|  | antisense: | 5'-GTCAAAGGCTAGAACATGCAGAC-3' |
| GAPDH | sense: | 5'-GCACCGTCAAGGCTGAGAAC-3' |
|  | antisense: | 5'-TGGTGAAGACGCCAGTGGA-3' |
| SNAIL | sense: | 5'-TTACCTTCCAGCAGCCCTAC-3' |
|  | antisense: | 5'-AGCCTTTCCCACTGTCCTC-3' |
| ZEB1 | sense: | 5'-AAGTGGCGGTAGATGGTA-3' |
|  | antisense: | 5'-TTGTAGCGACTGGATTTT-3' |
| TWIST1 | sense: | 5'-CGACGACAGCCTGAGCAACA-3' |
|  | antisense: | 5'-CCACAGCCCGCAGACTTCTT-3' |
| SLUG | sense: | 5'-CCTCCATCTGACACCTCC-3' |
|  | antisense: | 5'-CCCAGGCTCACATATTCC-3' |
| U6 probe (Life Technologies) | | |
| mir-455-3p probe (Life Technologies) | | |
